# Lethal outcome of Covid-19 pneumonia in a new liver recipient with neurological manifestation 

**Published:** 2020

**Authors:** Pegah Eslami, Muhammadhosein Moradi, Arash Dooghaie Moghadam, Ali Pirsalehi, Shabnoor Abdul Lateef, Amirreza Hadaegh, Behandokht Rezai, Amir Sadeghi, Hamid Asadzadeh Aghdaei, Mohammad Reza Zali

**Affiliations:** 1 *Gastroenterology and Liver Diseases Research Center, Research Institute for Gastroenterology and Liver Diseases, Shahid Beheshti University of Medical Sciences, Tehran, Iran. *; 2 *Student Research Committee, School of Medicine, Shahid Beheshti University of Medical Sciences, Tehran, Iran*; 3 *Department of Radiology, Amiralam Hospital, Tehran University of Medical Sciences, Tehran, Iran*

**Keywords:** Covid-19, Liver, Transplantation, Liver transplantation, Neurological complications

## Abstract

COVID-19 is a new contagious viral pneumonia with various signs and symptoms, including loss of consciousness, liver injury, and cerebrovascular accident; however, there is little data on the manifestation and outcome of COVID-19 in liver transplant patients. Moreover, because transplant units in Iran were closed from the first day of the COVID-19 pandemic, accurate data about nosocomial COVID-19 and the liver transplant setting is not available. In this article, we introduce a liver transplant recipient with a final fatal outcome, who had had neurological manifestations, and whose COVID-19 manifestations began in the hospital within 2 days of transplant surgery.

## Introduction

 On February 19, 2020, the Iranian Ministry of Health announced the first confirmed case of COVID-19 in Iran (1), approximately two months after the new pneumonia outbreak in Wuhan City, which spread surprisingly through China (1, 2). As of July 5, 2020, more than 237,000 confirmed cases and 11,400 deaths were recorded in Iran according to the 110th situation report, published by the World Health Organization (3).

The published literature has reported that COVID-19 patients have a broad spectrum of signs and symptoms, including fever, shortness of breath, anosmia, myalgia, and neurological involvement (4). The neurological manifestation of COVID-19 includes dizziness, loss of consciousness, headache, and cerebrovascular involvement (5). Moreover, other studies have shown that liver injury is frequently observed in COVID-19 patients, although limited information exists with regard to the liver transplant setting (6, 7). Nonetheless, the transplant society has encountered a number of problems during this pandemic. Since February, 2020, numerous transplant centers in Iran have decided to stop operations based on the primary data indicating that immunocompromised patients and patients with comorbidities are exposed to a higher risk of COVID-19 mortality and morbidity compared to other members of the society (8). Accordingly, the closing of operation rooms caused a further lack of knowledge about the impact of COVID-19 on new liver transplant recipients.

Presently, few studies have focused on the impact and manifestation of COVID-19 in liver transplant patients transplanted during the COVID-19 pandemic. Hence, describing clinical signs in liver transplant patients is crucial. Herein, we present a liver transplant recipient case who eventually died of COVID-19 in our hospital. 

## Case report

On January 28, 2020, a 69-year-old man suffering for eight months from chronic kidney disease and liver cirrhosis caused by Hepatitis C was admitted for liver transplantation. Upon admission, his medical history was examined and a physical examination was performed, revealing a temperature of 37.1 ℃, respiratory rate of 17, pulse rate of 84, and blood pressure of 130/90 millimeter Hg. The patient was diagnosed with ascites. No other significant gastrointestinal or hepatic findings such as abdominal pain, vomiting, nausea, diarrhea, or jaundice were reported. The patient received cadaveric liver transplantation according to the transplantation protocols in our hospital. Next, he was transferred to the ICU for further care before the temporary shutdown of surgical wards due to the COVID-19 outbreak in Iran.

**Table 1:** Laboratory data and vital signs alterations during hospitalization

Date	PLT*	BP**	WBC***	O2 Sat	INR****
Day 1	93	140/90	5.1	99.0	
Day 2	94		5.1	85.5	
Day 3	97		11.1		1.85
Day 4	98		14.2	99.1	1
Day 5			15.7	98.3	1.23
Day 6	66				
Day 7					
Day 8				99.0	1.20
Day 9	57				
Day 10		208/125		90.2	1.13
Day 11			7.5	85.2	
Day 12					
Day 13		98/58			1.11
Day 14			5.0	91.6	1.11
Day 15	34				
Day 16			4.8	78.7	1.11
Day 17	40		4.3		
Day 18			4.4		1.29
Day 19	29	90/60	4.4	90.3-98	1.24
Day 20	26	120/60			
Day 21		107/85			
Day 22		80/60			

*platelet, **blood pressure, ***white blood cells, ****International Normalized Ratio

On day 2 following transplantation, the patient showed a mild fever followed by mild dyspnea, which improved after non-invasive ventilation. Chest radiographic findings revealed a bilateral diffuse infiltration. The laboratory results indicated a white cell count of 5.1 × 109 /L, aspartate transaminase of 122 U/L, hemoglobin of 6.6 gram/deciliter, platelet of 94,000, creatinine of 2.3 milligram/deciliter, alanine transaminase of 110 U/L, overall bilirubin of 4.2 milligram/deciliter, and direct bilirubin of 2.5 milligram/deciliter. Blood gas analysis showed a PaO2 of 47.4 mmHg, a PaO2/FiO2 of 225.7, and an O2 saturation of 85.5%.

**Figure 1:**
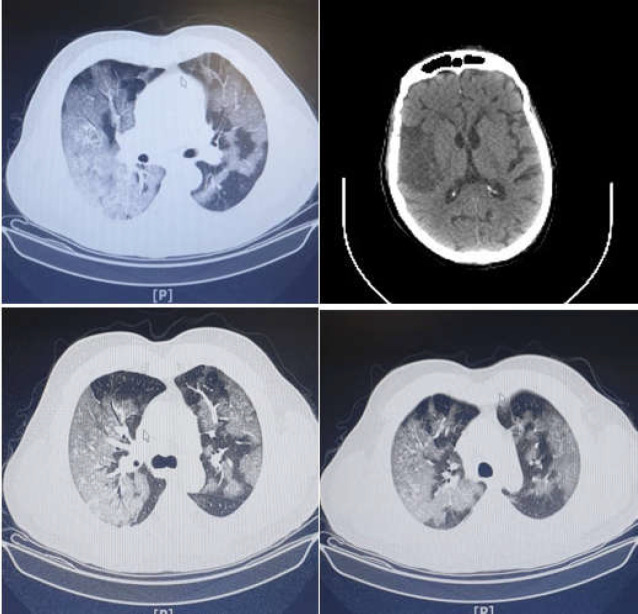
Chest and head CT scans performed during hospitalization

On day 4, the patient became febrile, and bilateral opacities along with left lung pleural effusion were observed in chest radiography. Thus, he was diagnosed with nosocomial pneumonia. Imipenem, meropenem, cotrimoxazole, and colistin were initiated and pleural TAP was performed. The patient received Tacrolimus and methylprednisolone according to the liver transplantation protocols. The respiratory status of the patient worsened and respiratory failure developed. The patient received invasive ventilation treatment. On day 9, the patient experienced loss of consciousness and had a GCS of 8. On day 12, oxygen saturation dropped to 50%, and the patient was intubated accordingly. Blood cultures were taken, and septicemia with pseudomonas species sensitive to ciprofloxacin and levofloxacin was detected. As a result, on day 15, a radiologic study of the patient revealed bilateral multilobar diffused ground glass opacities, which expanded to more than 70% of lungs interstitium (Figure 1). A CT scan of the patient’s brain was performed due to his lack of consciousness. The results showed a hypodensity in the right parietal lobe with gray and white matter involvement, suggesting an infarction of the middle cerebral artery, which could be due to acute or sub-acute brain infarction (Figure 1).

**Diagram 1:**
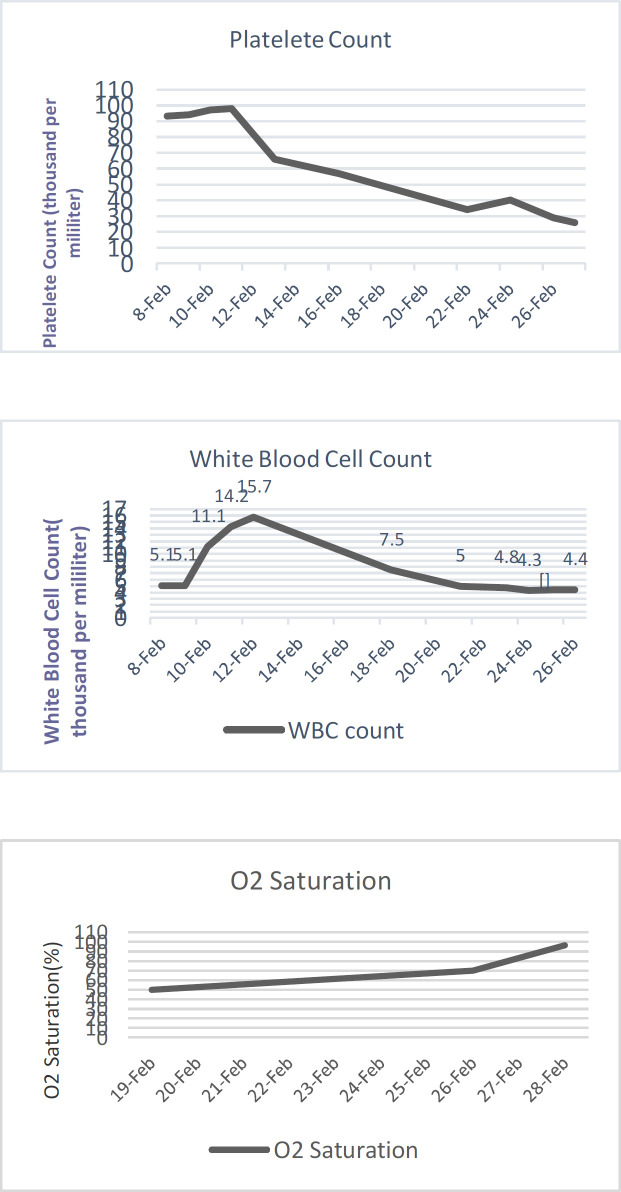
Laboratory data alterations during hospitalization

Sixteen days after transplantation, upon the announcement by the Ministry of Health and Medical Education (MOHME) regarding the outbreak of COVID-19, the disease was considered as a source of pneumonia as well. On day 19, the patient experienced another episode of LOC followed by a drop in O2 saturation to 70% and tracheostomy. The patient received continuous renal replacement therapy (CRRT) due to BUN/Cr rise during the disease. The radiographic X-ray findings of the white lung also appeared on the nineteenth day along with sepsis and anuria, indicating the side effects of CRRT (Table 1; Diagram 1). There was a decline in blood hemoglobin levels through the course of the disease, and the patient received pack cell transfusions accordingly. 

His platelet levels also dropped from 93,000 on day 1 to 20,000 on day 23. The patient’s health condition deteriorated on day 22. On day 23 after transplantation, a central pulse could not be detected, the patient’s O2 saturation level fell, and he became asystolic. After 45 minutes of several resuscitation attempts, the patient’s condition did not improve and he expired. A day after our patient died, his COVID-19 PCR was reported positive. 

## Discussion

Liver recipients are at high risk of community or hospital-acquired COVID-19 infection during the pandemic (9). Recipients in the incubation period of COVID-19 are at increased risk for post-surgery mortality and multi-organ damage after transplantation (9). Therefore, recognizing COVID-19 patients before transplant plays a pivotal role in decreasing hospital transmission and mortality (10). In several transplant centers, liver transplantation is performed only in urgent cases during a pandemic. In addition, all liver transplant candidates and donors should be checked for COVID-19 (10, 11). 

In our presented case, a fever began after the transplant and rapid radiologic findings similar to those reported for COVID-19 appeared. Ground-glass opacity showed the pulmonary alveoli to be partly filled with fluid. This radiologic finding has been seen in approximately 85% of COVID-19 patients. This finding can be accompanied by consolidation in about 60% of COVID-19 patients. Therefore, these findings are one of the most common radiologic features in such patients (12, 13). Unfortunately, due to a lack of knowledge about the presence of COVID-19 in our country at that time, we continued our liver transplant activity without any pre-surgical check for COVID-19. In this case, perhaps hospital-acquired COVID-19 occurred, or perhaps the patient was in the incubation period of COVID-19 before the surgery. Accordingly, the separation of transplant patients from each other and testing for COVID-19 should be a routine protocol during the COVID-19 pandemic.

In the presented case, fever started rapidly after liver transplant and continued until death, and the chest CT-scan showed bilateral ground glass opacities. These rapid changes in the patient’s status may have been due to surgery stresses and high levels of immunosuppression therapy. The radiologic findings in our case are compatible with the meta-analysis conclusions reached by Bao et al., declaring that ground glass opacity involving bilateral lungs with peripheral distribution patterns was the most frequent CT-scan manifestation in COVID-19 patients (14). Furthermore, our patient showed a decrease in hemoglobin and platelet levels, which is consistent with previous studies. Lippi et al. previously reported that a low platelet level was associated with a higher mortality rate and severity of disease (15). Xu et al. suggested that platelet aggregation in the pulmonary system, destruction by the human immunity system, and the direct invasion of COVID-19 to bone marrow caused a decrease in platelet levels (16). In another study, Lippi et al. found that hemoglobin levels decreased in patients with severe COVID-19 (17). Conversely, our patient experienced a loss of consciousness, and his CT scan results indicated a sub-acute cerebrovascular accident , which is a rare but crucial manifestation in COVID-19 patients (18). Previous studies have shown that hemorrhagic, ischemic strokes, and CNS vasculitis can be considered COVID-19 manifestations (19). The hosts were infected through the spike glycoprotein of COVID-19. This part of the virus can bind ACE2 receptors. This type of receptor is expressed in the renal, cardiac, and pulmonary systems as well as in endothelial cells (20). Therefore, this virus can invade all those organ systems. Histologic studies have mentioned that induced vasculitis can also occur in those organs (21). This event reminded us that during the COVID-19 pandemic, all atypical presentations must be considered as COVID-19 to be rechecked and re-evaluated.

Finally, we clarified that in this case, due to a lack of knowledge about the spread of COVID-19 in Iran in those times and the absence of a travel history to China, we first approached our patient as a case of influenza or bacterial pneumonia. However, after the MOHME announcement about COVID-19, we changed our approaches and final diagnosis.

In sum, our data about COVID-19 in liver transplant patients, especially during post-op, was limited. In this case report, we presented a liver transplant recipient who showed COVID-19 manifestation quickly after liver transplant surgery. Thus, we recommend that all patients be evaluated for COVID-19 before transplantation, and isolation should be considered for all transplant patients. Moreover, in the case of atypical presentation in a liver transplant patient, COVID-19 should be at the top of our differential diagnosis list during the pandemic. The collection of additional information about COVID-19 in liver transplant recipients is required.

## Conflict of interests

The authors declare that they have no conflict of interest.
